# Nanomechanics of β-rich proteins related to neuronal disorders studied by AFM, all-atom and coarse-grained MD methods

**DOI:** 10.1007/s00894-014-2144-5

**Published:** 2014-02-22

**Authors:** Karolina Mikulska, Janusz Strzelecki, Wiesław Nowak

**Affiliations:** Institute of Physics, Faculty of Physics, Astronomy and Informatics, Nicolaus Copernicus University, Grudziadzka 5, 87-100 Torun, Poland

**Keywords:** Atomic force microscopy, β-rich domains, Coarse-grained simulations, Contactin, Fibronectin, Gō-like model, Mechanical stretching of proteins, Neurexin, Steered molecular dynamics

## Abstract

Computer simulations of protein unfolding substantially help to interpret force-extension curves measured in single-molecule atomic force microscope (AFM) experiments. Standard all-atom (AA) molecular dynamics simulations (MD) give a good qualitative mechanical unfolding picture but predict values too large for the maximum AFM forces with the common pulling speeds adopted here. Fine tuned coarse-grain MD computations (CG MD) offer quantitative agreement with experimental forces. In this paper we address an important methodological aspect of MD modeling, namely the impact of numerical noise generated by random assignments of bead velocities on maximum forces (F_max_) calculated within the CG MD approach. Distributions of CG forces from 2000 MD runs for several model proteins rich in β structures and having folds with increasing complexity are presented. It is shown that F_max_ have nearly Gaussian distributions and that values of F_max_ for each of those β-structures may vary from 93.2 ± 28.9 pN (neurexin) to 198.3 ± 25.2 pN (fibronectin). The CG unfolding spectra are compared with AA steered MD data and with results of our AFM experiments for modules present in contactin, fibronectin and neurexin. The stability of these proteins is critical for the proper functioning of neuronal synaptic clefts. Our results confirm that CG modeling of a single molecule unfolding is a good auxiliary tool in nanomechanics but large sets of data have to be collected before reliable comparisons of protein mechanical stabilities are made.

**Figure:**
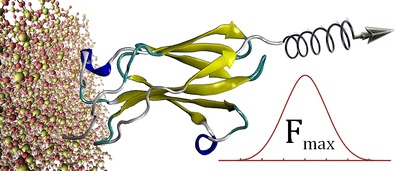
Computational strechnings of single protein modeules leads to broad distributions of unfolding forces

Computational strechnings of single protein modeules leads to broad distributions of unfolding forces

## Introduction

Atomic force microscopy (AFM) is a beautiful and powerful technique which enables single-molecule experiments [1]. AFM force spectroscopy helps to characterize physical properties of biological matter at the nanoscale [2]. However, experiments alone do not reveal the detailed molecular mechanisms leading to observed features in the force spectra (FS). The auxiliary information on conformational changes in biomolecules occurring during the forced unfolding (or unbinding) is obtained from carefully designed mutants [3] or from steered molecular dynamics (SMD) computer simulations [4–6]. In this variant of the molecular dynamics (MD) method an external force is attached to the molecule along a pre-selected coordinate [7] and this force is monitored with respect to time or elongation.

The mechanical properties of proteins are critical in numerous biological processes [3], for example, titin acting as an entropic spring helps to maintain the strength of muscles [8, 9], gankyrin is involved in cancer developments [10, 11]. A new field of mechano-enzymatics is quickly developing [12]. The stability of a neuronal synaptic cleft depends on proper structures of proteins present in the extracellular matrix. Detailed studies of biopolymer nanomechanics using both AFM and SMD have provided a better understanding of molecular design [13–17].

This opportunity for a synergy between theoretical modeling (SMD) and single-molecules experiments (AFM FS) is obscured by a mismatch between experimental and computational time scales. Experiments are typically 10^5^–10^6^ slower than typical ten nanosecond all-atom SMD simulations [18, 19]. Thus, the maximum forces predicted in computer unfolding experiments are a factor of ten higher than that measured by the AFM. To alleviate this problem coarse-grained (CG) SMD [20, 21] and Monte Carlo [22] methods have been proposed. Such an approach, for instance based on Gō-like models [23, 24], has been used in detailed studies of FnIII fibronectin domains, the I27 domain of titin protein, ubiquitin [21, 25]. Sulkowska and Cieplak used this very fast approach to determine strengths of 8000 subset of single domain proteins deposited in the Protein Data Bank (PDB) [26] and later even have enlarged the set of studied systems [27]. Despite its growing role in protein mechanical unfolding modeling only a few comparisons of CG approaches with respect to all-atom SMD have been published so far [21, 25]. The CG calculated unfolding path may be disturbed by fine details of the simulation protocol. The issue of unfolding force distributions, affected by numerical noise during CG stretching, has not been addressed yet. Thus, we performed extensive (nearly 2000 runs for each system) CG simulations for a set of β-rich model proteins using a Gō-like model introduced by Karanicolas and Brooks [23, 24]. The proteins in this set have an increasing complexity. In addition, all atom (AA) SMD simulations were performed for the same systems.

It is known, that the mechanical strength of proteins depends to a large extent on their secondary structure composition. β-strands, linked by numerous hydrogen bonds, are perhaps the main reason of high mechanical stability of typical protein folds [16, 19, 28–30]. As model systems we have selected fragments of proteins important in the maintenance of synapse functions: contactin (CNTN), fibronectin (FN), and neurexin (NRXN). Aberrations in the contactin gene affect proper connections between pre- and postsynaptic neurons and may lead to autism spectrum disorder [31]. Similarly, nanomechanics of the pre-synaptic protein neurexin is crucial for maintaining its shape. The correct shape of **NRX** is critical for proper interactions with the postsynaptic adhesive protein neuroligin (NLG) [32]. A large modular protein, fibronectin, is present in the extracellular matrix of neurons: it plays a role in wound healing, and is considered as a possible marker of cancer [33].

In this paper we compare average maximum unfolding forces, force distributions and mechanical unfolding paths of a short β-peptide (**f**–**H**) present in fibroin, two Greek key β-sandwich domains: one from CNTN4 (FNIII_3_ domain) and one from **FN1** (FNIII_9_ domain) and a complex structure – the LNS_5_ domain of NRXN1α. The results were obtained from CG SMD and AA SMD simulations. Some new experimental AFM spectra from our measurements of CNTN, **FN1**, and NRXN stretching are presented here as well. Clear differences between nanomechanics of varying β-structures revealed by CG stretching and a more detailed AA modeling approach are observed. However, the fast CG modeling of the AFM experiments gives a correct range of forces and deserves further development and improvement. Our data indicate that it has a great potential for wide applications in the computational design of new materials and nanoscience.

## Materials and methods

**Fig. 1:**
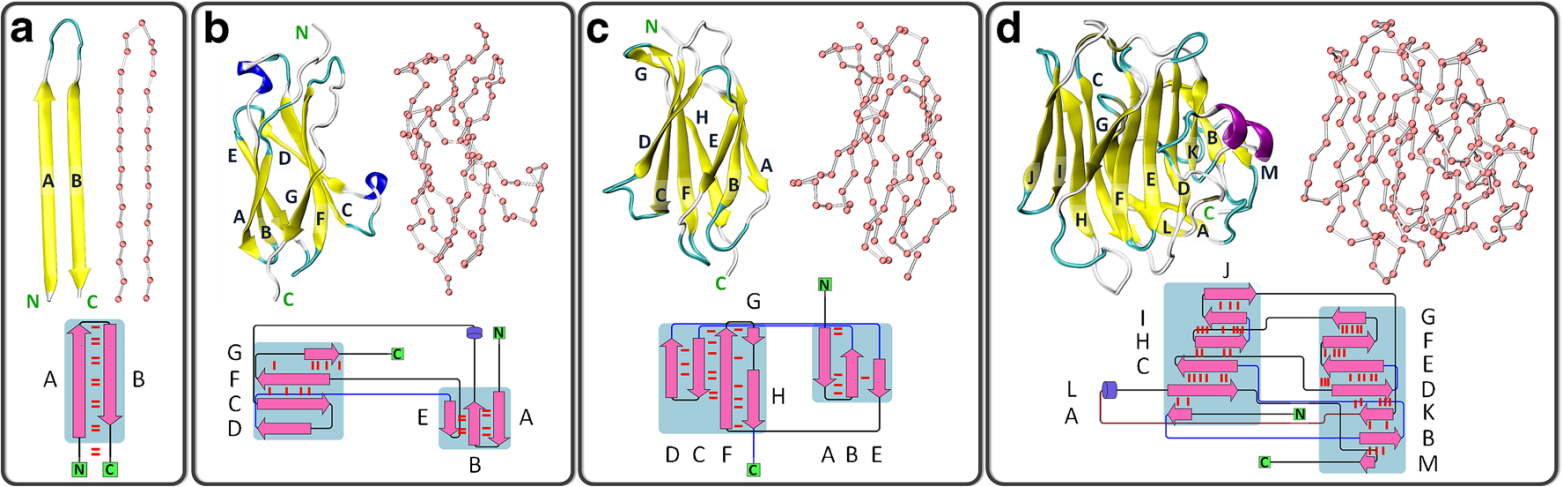
The initial all-atom and coarse-grained structures of the β-rich protein domains and their topologies: **a** Fibroin-H motif (**f**–**H**), **b** FnIII_3_ CNTN4 (**CNT**), **c** FnIII_9_ of fibronectin 1 (**FN1**), **d** LNS_5_ of NRXN1α (**NRX**). The figure was prepared using the VMD program [34] and Pro-origami server [35]

The mechanical properties of four molecular systems having an increasing number of β strands were investigated: a fibroin-H fragment (**f**–**H**), FnIII_3_ a domain of contactin4 (**CNT**), an FnIII_9_ domain of fibronectin1 (**FN1**) and an LNS_5_ domain of neurexin1α (**NRX**). The folds and topologies of these systems, i.e., the initial AA and CG structures, are shown in Fig. 1. The coordinates for the proteins studied by the AFM method (**CNT**, **NRX**) were adopted from the PDB structures of similar organisms (see in Table 1).

**Table 1:** β-rich protein domains studied in all-atom and coarse-grained SMD simulations

Acronym	Name of β-rich protein domain	Number of amino acids	Template structure	Organism/protein of the template structure
**f–H**	Fibroin-H fragment form *Trichoptera*	31	UniProtKB sequence (a.n.: A5A6G5)	*Trichoptera* Fibroin-H
**CNT**	Human FnIII_3_ CNTN4	102	2ee2	Human FnIII_3_ CNTN1
**FN1**	Human FnIII_9_ FN1	92	3t1w	Human FnIII FNI
**NRX**	Rat LNS_5_ NRXN1α	173	3asi	*Bos Taurus* LNS_5_ NRXN1α

### All-atom steered molecular dynamics (AA SMD) simulations

AA protein models were solvated using 0.7 nm layer of the TIP3P water model [34] in each dimension. A concentration of 150 mM NaCl was maintained in each system. In all MD simulations a cutoff of 12 Å for non-bonded interactions was applied. Langevin dynamics and Langevin piston algorithms were used to maintain temperature at 300 K and pressure at 1 atm. The multiple time step method was employed as implemented in the NAMD 2.8 code [35], with time steps of 1 fs for bonded, 2 fs for short-range non-bonded, and 4 fs for long-range electrostatic forces. AA trajectories were computed using the all-atom CHARMM27 force field [36]. In preparatory simulations we performed the following steps: 0.2 ns of water equilibration, 10,000 steps of minimization, 0.35 ns of heating from 0 K up to 300 K and 0.15 ns equilibration of the whole system before each “production” SMD simulation. The constant velocity SMD method was used to stretch all structures along a N-C vector. The N-C vector connected the C_α_ atoms of the N- and C-terminal residues at positions found in the trajectory after the water equilibration step. In the SMD technique a virtual harmonic force is applied to one end (N-terminus) of the protein whereas the other end (C-terminus) is simultaneously fixed. The structures were stretched at a constant speed of 0.025 Å/ps with a spring constant K of 4 kcal (mol  Å^2^)^−1^ (278 pN/Å). One should note that such protocol results in a varying force acting on the protein: the larger a distance D between the pilot “dummy atom” from the pulled atom, the larger the force acting on the protein. The current force (in pN) is calculated from the formula:

1 $$ \mathrm{F}=\mathrm{K}\ast \left(\mathrm{D}-{\mathrm{D}}_0\right), $$

where D_0_ denotes the initial, i.e., starting, frame distance. When a sudden conformational transition leading to a more extended conformation (mechanical unfolding) occurs the distance D between the pilot “dummy atom” and the pulled atom decreases and the force drops down.

We have run five (4–25 ns) AA SMD simulations of each system (6000 to 14,200 atoms) studied, thus the total time was over 300 ns. The Visual Molecular Dynamics (VMD) software [37] (version 1.9.1) and home-made scripts were used to analyze output trajectories.

### Coarse-grained steered molecular dynamics (CG SMD) simulations

CG SMD trajectories were computed with the help of CHARMM [38] software using Gō-type model [39] of proteins. Each protein model and topology, as well as parameter files, were generated from the corresponding β-rich all-atom structure using the MMTSB web server [24]. The C_α_ model proposed by Karanicolas and Brooks [23, 24] was employed. In this model a protein is represented by a series of C_α_ pseudo-atoms, linked by properly tuned harmonic springs. Each bead has a mass of the corresponding amino acid. The potential energy surface is constructed using fine-tuned rules oriented toward good reproduction of folding processes. Native hydrogen bonds are taken into account. The interaction energies of pseudo-atom pairs separated in sequence by three or more bonds were approximated as a modified Lennard-Jones function [23]:

2 $$ {V}_{ij}={\varepsilon}_{ij}\left[13{\left(\frac{\sigma_{ij}}{r_{ij}}\right)}^{12}-18{\left(\frac{\sigma_{ij}}{r_{ij}}\right)}^{10}+4{\left(\frac{\sigma_{ij}}{r_{ij}}\right)}^6\right], $$

where r_ij_ is the distance between “residues” *i* and *j*, σ_ij_ is the distance between *i* and *j* at which the interaction energy is a minimum, and ε_ij_ is the depth of the potential well for the pseudo-atom pair *ij* at this distance. In addition, in the total CG force field terms corresponding to hydrogen bonds and sequence dependent torsional angles are also present, for details see [23] and [24].

The temperature was maintained at 300 K using a Langevin thermostat. Since in each CG SMD trajectory velocities corresponding to this temperature were randomly assigned, that process generated numerical noise. The time step of 10 fs was used and holonomic constraints were applied to the C_α_ bonds. The CG method is very efficient but as many simulation protocols may depend on fine details of initial conditions and numerical noise, in order to study possible force distributions, we have run over 2000 CG implicit solvent SMD simulations for each structure (Fig. 1), using the same initial conditions. In constant velocity CG SMD simulations a harmonic spring with 100 pN/Å spring constant was added to the C-terminal of the studied models. The same pulling velocity as in the all-atom simulations (0.025 Å/ps) was used. Moreover, in order to monitor qualitative dependence of forces on the pulling speed, we generated 1000 CG SMD trajectories for **CNT** with the pulling velocity ten times slower (0.0025 Å/ps) and 20 CG SMD simulations with a very low pulling velocity of 0.001 Å/ps (five simulations for each studied domain, 0 K initial structure). Total simulation time of all our CG SMD trajectories was 26.6 μs. Home-made scripts were used to analyze outputs and CG SMD trajectories.

## Results and discussion

### CG SMD stretching of four β-rich protein modules

**Fig. 2:**
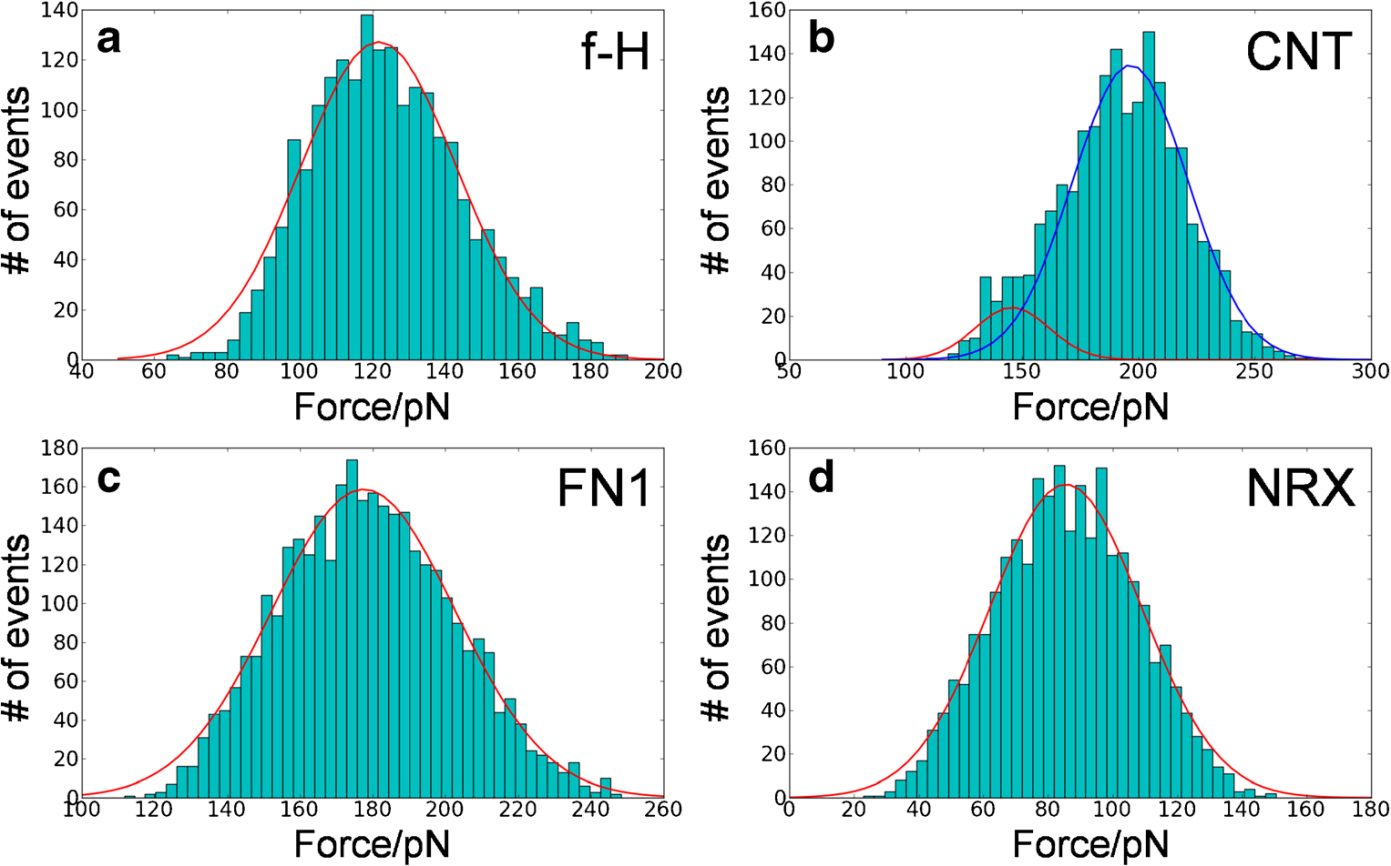
Maximum force histograms as established in CG SMD simulations (each data set contains at least N >2000 simulations)

We have studied β-rich systems with increasing complexity: a very simple one (an **f-H**, fibroin-H protein fragment with only two β stands), two medium size typical modules (**CNT** and **FN1** both have 7 β strands in two β-sheets), and a complex one (**NRX** has 13 β stands in two β-sheets). To the best of our knowledge, **NRX** is the largest system studied using the present Gō-like SMD model so far. We have registered force-extension (or force-time) stretching curves. The maximum forces from each of these spectra were extracted and recorded. For the basic pulling speed of 0.025 Å/ps more than 2000 maxima were collected for each system studied. Different maximum forces resulted from statistically different unfolding trajectories. The nature of distribution of forces helps in the interpretation of SMD/AFM experiments and is studied here. A simple Gaussian function was fitted to the force maxima distributions (see Fig. 2). The maxima of these Gaussian distributions, standard deviations, protein modules extensions at the maximum force and the corresponding time points are collected in Table 2.

**Table 2:** Averaged maximum force (F_max_), distance between fixed and pulled atom (r_NC_), time registered at the maximum force (t) from CG SMD simulations (*v* = 0.025 Å/ps). Simulation times were 0.6 ns, 1.4 ns, 1.4 ns, and 3.2 ns for **f-H**, **CNT**, **FN1**, and **NRX** respectively

Protein	F_max_ [pN]	r_NC_ [nm]	r_NC_^*native*^ [nm]	t [10^−1^ ns]
**f–H**	121.7 ± 21.4	5.51 ± 0.10	0.54	2.08 ± 0.03
		6.11 ± 0.16	0.54	2.31 ± 0.06
		8.17 ± 0.11	0.54	3.14 ± 0.04
**CNT**	145.8 ± 15.9	6.87 ± 0.08	4.55	1.02 ± 0.03
	196.5 ±24.8	15.11 ± 0.13	4.55	4.31 ± 0.06
**FN1**	177.1 ± 25.3	4.40 ± 0.06	4.21	0.16 ± 0.03
		4.61 ± 0.14	4.21	0.26 ± 0.03
**NRX**	85.5 ± 23.5	10.22 ± 0.35	1.52	3.45 ± 0.15
				3.62 ± 0.06

The registered maximum forces vary from about 90 pN (**NRX**) to nearly 200 pN (**CNT**). These values are in very good agreement with numerous AFM experiments where similar modules were stretched [13, 28, 40, 41]. Earlier results, obtained by the same Gō-like CG model by E. Paci for I27 yielded values between180 and 250 pN [21]. One may correlate the mechanical strength determined here by the CG simulations with the number of H-bonds present in the initially pulled flanking β stands. We observe that these mechanical barriers are the highest in **CNT** and **FN1** where around 10-11H-bonds may be distinguished. In the **f-H** model we have 13H-bonds and even this very simple fragment requires a relatively high force to be ruptured. A rather low F_max_ of 90 pN is observed for the largest domain of **NRX**. We attribute this observation to two factors: (a) weak coupling between the adjacent β stands (see Fig. 1 - the largest number of connecting H-bonds is only five) (b) a relatively long MD simulation time. Before this large structure of **NRX** starts its effective unfolding, a long period of time is needed for a proper reorientation and the protein has ample time for “finding” such a low force unfolding path. When five times higher pulling speeds are applied to **NRX**, the recorded unfolding forces increase to 120–150 pN (unpublished results).

The widths of fitted Gaussian distributions of forces (see Table 2 and Fig. 2) are similar for all protein modules, but not identical. We show that CG simulations ran using the same initial conditions may give quite distinct maximum forces with standard deviations as large as 20–25 pN. In Fig. 2 histograms of CG maximum forces are presented. It is noteworthy that these distributions are rather wide. In each individual case the direction of the pulling force was always the same, but the values of the recorded maxima, due to statistical character of the unfolding process, vary in a quite wide range of  ± 20 pN.

One should note that the maximum mechanical unfolding force may depend on the points where the protein is stretched and on the direction of the force, as formally discussed by Kumar et al. [42, 43]. SMD modeling studies using protocols similar to those used here have already noticed such dependence of a maximum force on a direction of the pulling force vector [21, 44]. This phenomenon is related to a very complex energetic landscape of proteins [18, 42, 43, 45] and a particular scenario of hydrogen bonds rupture. For example, uncoupling two β-strands connected by numerous hydrogen bonds proceeds through distinct mechanisms for “parallel” and “perpendicular” pulling forces [21]. Usually a rather low force is sufficient for sequential breakage of the bonds (a “perpendicular” force leads to sequential unzipping of β-strands) while very high resistance is met when concerted breakage occurs (a “parallel” force, particularly strong hydrogen bonds force clamp is observed in parallel β-sheets). In our SMD simulations we always kept the pulling force vector along the NC axis, however, a relative orientation of this pulling force with respect to a locally formed force clamp made of adjacent β strands may vary from case to case.

Our data clearly show that the interpretation of AFM experiments based on just a few CG simulations/trajectories may be prone to a 10 % error in the maximum forces calculated. The studies of forced unbinding [46] in drug design should also take this observation into account, before a recommended procedure for prospective drugs undocking is established.

An extension at maximum force registered in CG SMD simulations gives approximate information where the force clamp for each β-type module is located. From the data indicated in Table 2 one can see that maxima are observed at initial or middle phases of the stretching processes. We estimate that CG force maxima occur at 1–35 % of the full stretch length of **CNT**, **FN1**, **NRX**. In a small **f-H** peptide the maximum is in the middle of the full unfolding path.

**Fig. 3:**
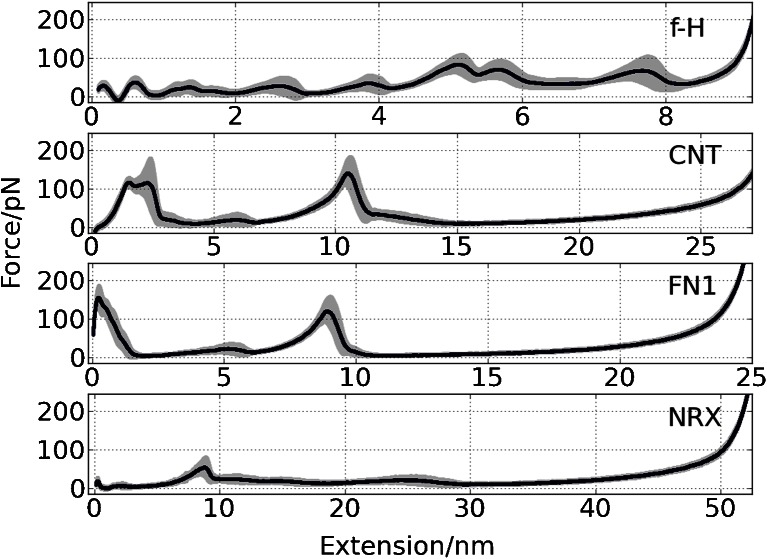
Averaged curves with standard deviations (*in gray*) for each model obtained from 500 random CG SMD simulations (*v* = 0.025 Å/ps)

Particularly interesting data are gathered for the **CNT** module (see Fig. 2b, Table 2). The distribution of maximum forces is the best approximated by two Gaussian functions. Force clamps occur not only at the initial phase of the unfolding but at 40 % of the total length as well. This demonstrates that two distinct groups of maxima in **CNT** unfolding paths exist. A further analysis should reveal whether numerous unfolding paths are predicted here. It seems that the **FN1** protein module also has two intermediates with high mechanical resistance (see Fig. 3); however, the first maximum dominates, thus just one Gaussian function correctly describes calculated maximum forces for **FN1** (Fig. 2c). CG data indicate that **NRX** is relatively prone to mechanical unfolding – forces required are lower (85.5 ± 23.5 pN) than those observed for other β-systems studied here. The extension at the maximum force weakly depends on the pulling atom speed.

In Fig. 3 we compare 500 individual CG force spectra for each model studied. As a narrow span of a standard deviation line indicates the spectra are quite self-similar showing that FS characteristics of individual domains are not affected much by numerical noise. One should note that these data do not exclude the possibility of alternative mechanical unfolding paths.

In earlier papers on early Gō-like models some criticism was raised against the selectivity of these simple approaches as far as force spectra are concerned, especially in topology-only models [47]. Protein structures of similar size exhibited very similar, non-specific, unfolding paths. Fortunately, the model used here is more elaborated [23, 24], and therefore all our force spectra are distinct, even for the closely related **CNT** and **FN1** pair (Fig. 3).

### All-atom SMD stretching of four β-rich proteins

**Fig. 4:**
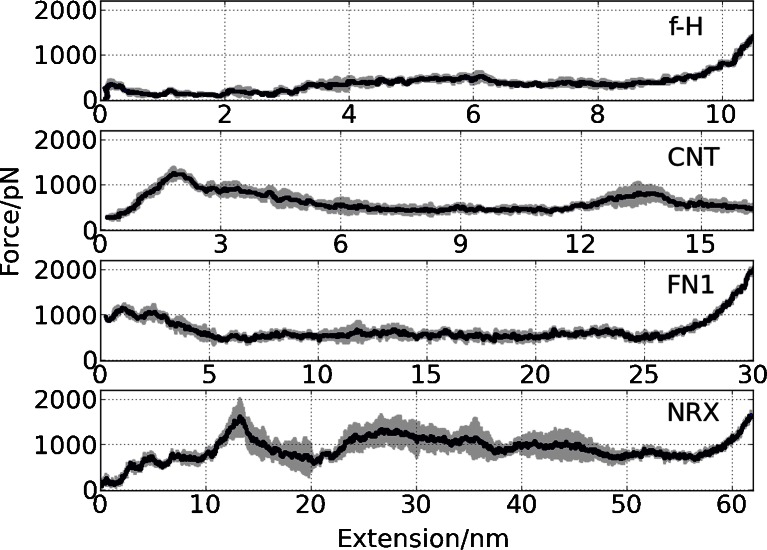
Averaged curves with standard deviations (*in gray*) for each model obtained from 5 AA SMD simulations (*v* = 0.025 Å/ps)

The averaged AA SMD force-extension curves are located at much higher forces (see Fig. 4) than those for CG SMD (Fig. 3). The spectra are specific to each protein studied. Closer inspection of these figures suggests that **NRX** perhaps exhibits alternate AA SMD unfolding paths (large variation in AA FS spectrum indicated by gray area in Fig. 4). The local maxima on each of our AA FS curves may be easily interpreted using computer graphics. We were curious whether CG models reflect similar features of mechanical unfolding spectra and to what extent that simplified model is capable of indicating mechanically stable intermediates.

**Fig. 5:**
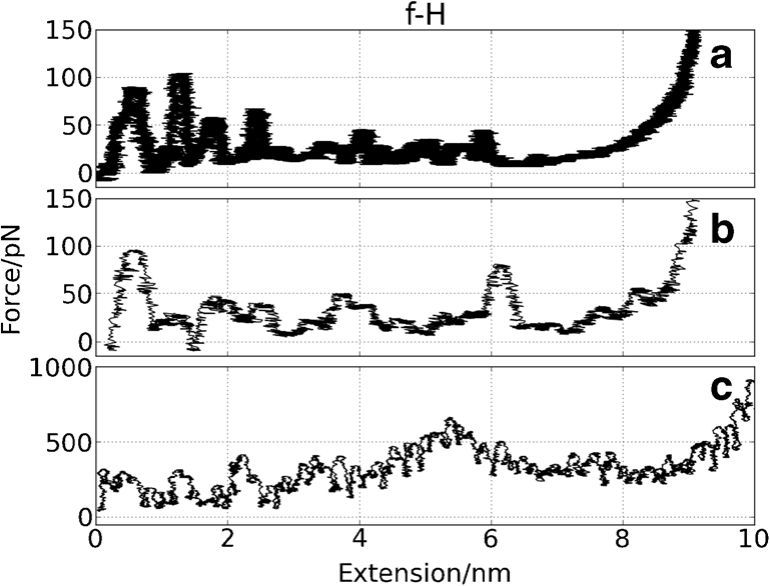
Force vs. extension curves for **f–H** from: **a** CG SMD (*v* = 0.001 Å/ps), **b** CG SMD (*v* = 0.025 Å/ps), **c** all-atom SMD simulation (*v* = 0.025 Å/ps). Running averages were used

### A comparison of CG SMD spectra with all-atoms spectra

**Fig. 6:**
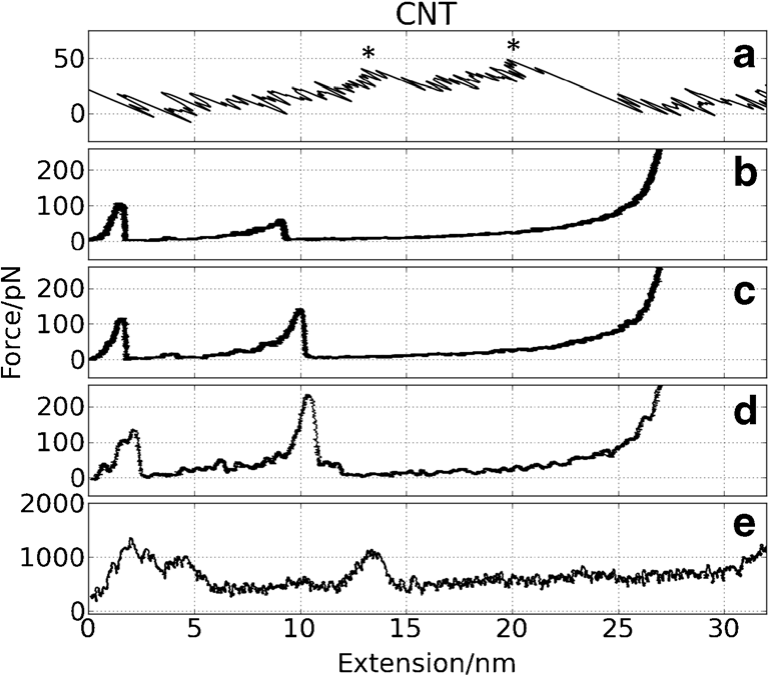
Force vs. extension curves for CNT from: **a** AFM measurements, **b** CG SMD (*v* = 0.001 Å/ps), **c** CG SMD (*v* = 0.0025 Å/ps), **d** CG SMD (*v* = 0.025 Å/ps), **e** all-atom SMD simulation (*v* = 0.025 Å/ps). Running averages for simulation curves were used. Two maxima in AFM spectrum are indicated by *stars*

**Fig. 7:**
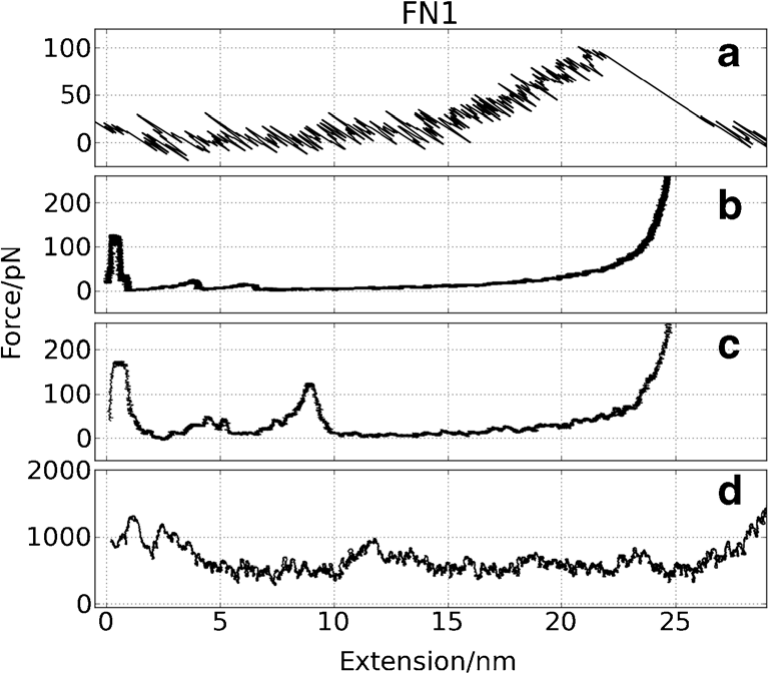
Force vs. extension curves for FN1 from: **a** AFM measurements, **b** CG SMD (*v* = 0.001 Å/ps), **c** CG SMD (*v* = 0.025 Å/ps), **d** all-atom SMD simulation (*v* = 0.025 Å/ps). Running averages for simulation curves were used

**Fig. 8:**
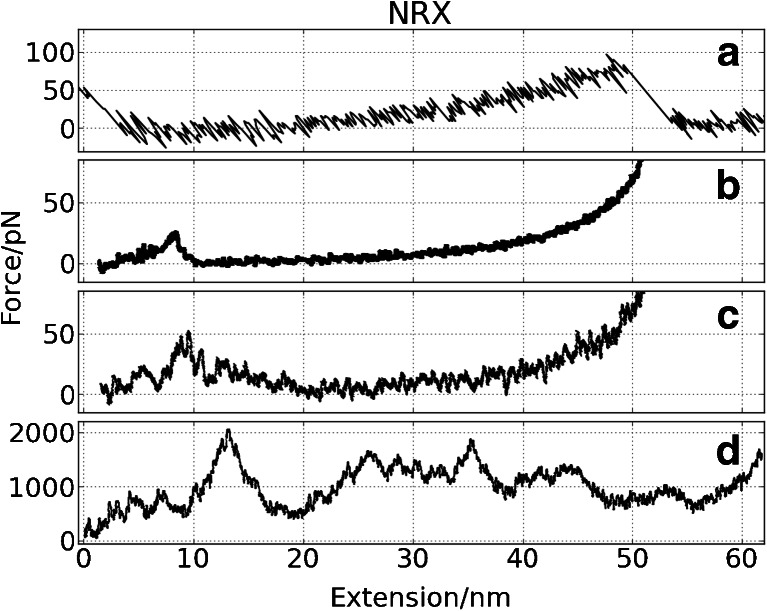
Force vs. extension curves for NRX from: **a** AFM measurements, **b** CG SMD (*v* = 0.001 Å/ps), **c** CG SMD (*v* = 0.025 Å/ps), **d** all-atom SMD simulation (*v* = 0.025 Å/ps). Running average for simulation curves were used

We have tried to correlate qualitative features of CG FS spectra with AA force spectra calculated using the same pulling speed. One can see that in general AA spectra show different characteristics than that of CG models (Figs. 5, 6, 7, and 8).

### A comparison of CG SMD unfolding paths with all-atom SMD and AFM force spectra

Several studies discuss a discrepancy between AFM force spectra from single molecule pulling experiments and results of all-atom SMD simulations [48, 49]. So far only a qualitative agreement between AFM measurements and AA SMD has been obtained. Discrepancies in quantitative matching between a real experiment and the computational model have been a persistent problem. In fact, peak values of forces predicted by the all-atom SMD simulations were typically about ten-fold higher than the observed AFM values [48].

**Fig. 9:**
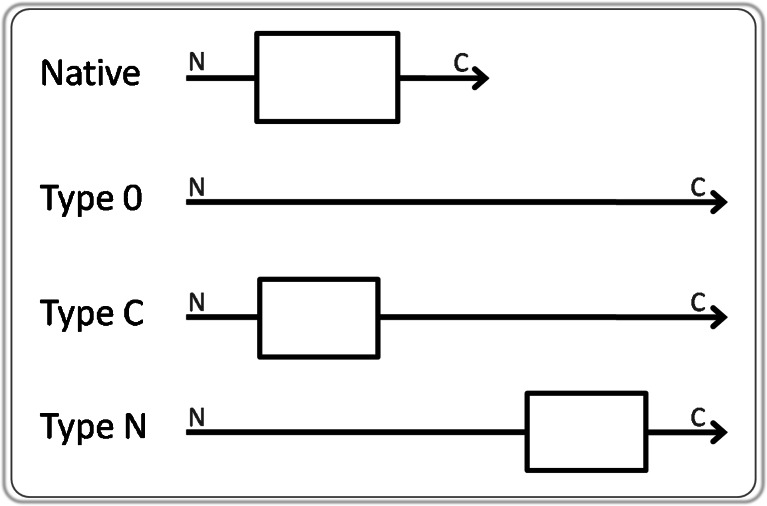
A rough classification scheme of mechanical unfolding scenarios. When protein modules are pulled by a force attached to the C-terminus, alternate paths are possible: a uniform unfolding without clear intermediates (type 0), a dominant unfolding at the C-terminus with an intermediate (or intermediates) located close to the N-terminus (type C), a similar scenario but with the N-terminus part unfolding at the initial stage (type N). On rare occasions unfolding happens symmetrically at both ends (type NC, not shown)

In order to facilitate a discussion of paths we introduce a rough classification of unfolding scenarios (see Fig. 9).

The analysis of spectra presented in Fig. 3 (CG) and Fig. 4 (AA) allowed for a rough classification of unfolding scenarios. The results are summarized in Table 3.

**Table 3:** Unfolding scenarios in CG and AA SMD simulations. Particular types are specified in Fig. 4

	CG SMD	AA SMD
f–H	NC/0	NC/0
CNT	C/NC/0	C/0
FN1	C/NC/0	NC/C/NC
NRX	NC/C/0	C/0

The general unfolding scheme predicted by both methods is similar, but not identical. In a small **f-H** system there are no intermediates and in both CG and AA approaches only the NC scenario is present. In **CNT** the initial unfolding starts from the C-terminus according to both methods. However, in a similar size **FN1** domain CG indicates that the C-terminal part is more prone to unfolding and AA SMD gives the opposite result. Similarly, in a very large **NRX** system, different pictures of initial unfolding phases are predicted by the CG and AA methods. In summary, our results indicate that qualitative scenarios of mechanical unfolding paths calculated by the CG model are not in 1:1 correspondence with those obtained using AA model of a protein module. One should remember that this classification is somehow simplified, since multiple unfolding paths are present in β-modules having more complex topologies.

Using computer graphics we have correlated the CG FS maxima with breaking particularly strong “force clamps” events:

#### f-H

In a simple **f-H** system we observe numerous maxima in the force (∼10, see Fig. 5a,b) which are related to hydrogen bond breaking events in AA SMD (Fig. 5c). H-bonds are only implicitly present in the CG model, but the shape of FS are qualitatively similar to that obtained in AA SMD. We observe, in accordance with earlier works [42, 43], that the detailed spectrum depends both on the pulling force direction and the pulling speed.

#### CNT

Some AFM spectra of **CNT** show double maxima denoted by stars in Fig. 6. CG and AA have two maxima as well; they are well separated by 8 nm of expansion length. The maxima correspond to intermediates I_1_ and I_2_ in mechanical unfolding paths of **CNT**: the I_1_ clamp appears during the detachment of stand G from F, and I_2_ corresponds to the breakage of B/E interface (see Fig. 1b). The natures of intermediates predicted by the AA and CG models are the same. Two maxima are registered in the AFM spectrum as well (Fig. 6a).

#### FN1

The CG FS of **FN1** (Fig. 7) are similar to those of **CNT** (Fig. 6). Here we also have two clear intermediates: I_1_ (H/F and G/F interfaces breaks, see Fig. 1c) and I_3_ (B/E linkage is ruptured). Between them the third small intermediate (I_2_) appears in a number of CG trajectories (related to A/B interface). In this system multiple CG unfolding scenarios were found. We did not observe multiple maxima in the AFM force spectra. In AA NAMD/CHARMM/TIP3P water simulations the unfolding scenario is consistent with results presented by Paci and Karplus [50] obtained with an implicit solvent, a CHARMM force field and the biased MD method. The sequence of events is similar to CG simulations but in AA I_3_ the F/C linkage is ruptured slightly faster than the B/E interface. In the AA data set we have also observed one distinct scenario where the unfolding of one end dominates, similar to that described for tenth module of fibronectin [51]. The presence of an intermediate state in Fibronectin type III modules has been discussed in several studies [52, 53]. Two maxima in FS of FNIII modules were observed in many previous simulations [50, 53, 54] as well.

#### NRX

In **NRX** CG FS only one relatively weak maximum dominates (Fig. 8b,c) – it is related to H/B and A/L force clamps (see Fig. 1d). The breakage of this region results in the NC phase of unfolding scenario. Later the C-term end of **NRX** is being unfolded and there the L/C interface splits apart. The AA SMD picture for **NRX** is different: mainly the C-term end is affected, and the folded core of **NRX** undergoes two substantial rotations (at 10 nm and at 20–25 nm) under the force resulted from the NC pulling.

The AFM spectra measured in our lab, using protocols described elsewhere [13] (see Figs. 6, 7, and 8), do not provide direct information on unfolding scenarios for short modules studied here. More elaborate experiments exploiting tandem repeats and engineered mutants are necessary. However, we note, that values of F_max_ calculated by the CG model are in a much better quantitative agreement with experiments than those from AA SMD modeling. The values of F_max_ are tuned by relative orientation of β-stands being ruptured at each unfolding step. It is necessary to note that the magnitude of the force depends not only on the number of H-bonds in the critical region, but also on the orientation of the pulling force with respect to the stretched protein fragment [42, 43, 45, 55]. Our simulations exploited only one arbitrary NC oriented force vector. Mechanical resistance of classical Ig-like folds, with parallel β-sheet, is usually higher than protein modules having antiparallel β-sheet [56]. In systems studied here mainly antiparallel adjacent β-stands generated sheer force clamps.

### Significance of nanomechanical behavior for neurological disorders

As we mentioned in the Introduction, mutations in **CNT** [31] or **NRX** [32] genes are linked with such diseases as ASD [57]. For example, contactins modulate neurite outgrowth, synaptogenesis and survival, also play a role in guidance and branching of axons in forming neural circuits [58]. Both proteins have large numbers (ten) of individual IgC2, FNIII, LNS or EGF modules, similar in size and composition to those studied here. The modified amino acid sequence, or lack of substantial portion of a protein, perhaps modulates the strength of interactions of **CNT** and **NRX** with partner proteins, such as a product of contactin associated protein-like 2 (CNTNAP2) gene or neuroligin in a synaptic cleft. One may expect, that even under physiological conditions, **CNT** and/or **NRX** undergo mechanical stress, for instance, under sudden acceleration of the organism, during injury or during formation of the nervous tissue and a protein transport across membranes. The nanomechanical resistance of proteins correlates with their function [8, 9, 12, 14, 59], for example, with mechanotransduction of signals. Our AFM measurements for **CNT** and **NRX** modules show that **NRX** is more mechanically stable than **CNT**. Relatively low forces are required for partial unfolding of this β-structure rich segments. Interactions of such stretched proteins with external factors (enzymes, components of extracellular matrix, neurotransmitters) are therefore modulated by even a low force. Intermediates in **CNT**, **FN1**, and **NRX** were identified in both AA and CG SMD simulations. It is tempting to speculate that the stressed **CNT** or **NRX** modules change interactions with partners, and in that way contribute to plasticity of a synapse and modify memory effects. Further studies on comprehensive sets of protein variants are necessary in order to estimate what aspects of protein mechanics are critical for good synapse development and functioning. The present study provides data for such comparative computational studies of medically important modular proteins.

## Conclusions

In this paper we presented comprehensive data on maximum forces calculated using CG model and the SMD method occurring during mechanical stretching of selected β-rich protein modules. It has been found that calculated CG FS maxima have values reasonably close to those measured with AFM. The maximum forces obtained from >2000 (31,000 ns) trajectories coupled by the same initial conditions exhibit Gaussian distribution with averaged values ranging from 86 ± 24 pN (**NRX**) to 197 ± 25 pN (**CNT**). The quantitative agreement between the CG force maxima with our experimental AFM data for **CNT**, **FN1**, and **NRX** was much better than those of SMD force spectra obtained with the CHARMM27 AA model. For the set of proteins studied here each force spectrum exhibits individual, specific features. Furthermore, main characteristic features which occur in the unfolding paths in AA SMD curves have counterparts in the CG SMD simulations. The final unfolding length of domains can be reached in AA SMD simulations much earlier that in the CG SMD simulations. This is probably due to the specific formulation of the present CG Gō-like model. We observe, in accordance with the previous reports (for example: [56], [44], Fig. 2b), that with decreasing pulling velocity the force required for protein unfolding decreases. The exact extrapolation of F_max_ values to velocities used in AFM experiments requires a separate study.

The topology of a protein can be a useful indicator of protein strength. The length of the strands at the N and C termini and the number of hydrogen bonds to neighboring strands are crucial factors for the protection of a system against mechanical stress. The topology of β strands (parallel or anti-parallel orientation) is also related to mechanical stability of protein modules [42, 43, 56]. Our observations are similar to previous SMD reports for titin [9]. Current results indicate that a large number of CG SMD simulations have to be performed in order to give meaningful and trustworthy data on the nanomechanical stability of protein modules.

## Abbreviations

AA: All-atom
ASD: Austism spectrum disorder
CGMD: Coarse-grained molecular dynamics
CNTN: Contactin
EGF: Epidermal growth factor like domain
FN: Fibronectin
FS: Force spectrum
IgC2: Immumolobulin like domain type C2
LNS: Laminin G domain
MD: Molecular dynamics
NRXN: Neurexin
NLG: Neuroligin
PDB: Protein data bank
SMD: Steered molecular dynamics
